# Parental psychiatric disorders and age-specific risk for offspring major depression: Finnish nationwide register-based study

**DOI:** 10.1017/S0033291725000662

**Published:** 2025-03-26

**Authors:** Subina Upadhyaya, Dan Sucksdorff, Miina Koskela, Keely Cheslack-Postava, Alan S. Brown, Andre Sourander

**Affiliations:** 1 Department of Child Psychiatry, Research Centre for Child Psychiatry, University of Turku, Turku, Finland; 2 INVEST Research Flagship, University of Turku, Turku, Finland; 3 Department of Psychiatry, Turku University Hospital, Turku, Finland; 4 Department of Child Neurology, Turku University Hospital, Turku, Finland; 5 Department of Psychiatry, New York State Psychiatric Institute, Columbia University Irving Medical Center, New York, NY, USA; 6 Department of Epidemiology, Columbia University Mailman School of Public Health, New York, NY, USA; 7 Department of Child Psychiatry, Turku University Hospital, Turku, Finland

**Keywords:** maternal, offspring, major depression, parental, psychiatric disorders, paternal, psychopathology, register-based, familial, age-specific risk, nested case-control, child

## Abstract

**Background:**

Limited studies have examined the association between the whole range of parental psychopathology and offspring major depression (MD). No previous study has examined this association by age of onset of offspring MD, or restricting to parental psychiatric diagnoses before offspring birth.

**Methods:**

This nested case–control study included 37,677 cases of MD and 145,068 controls, identified from Finnish national registers. Conditional logistic regression models examined the association between parental psychopathology and MD, adjusting for potential confounders.

**Results:**

Increased risk of MD, expressed as adjusted odds ratio and 95% confidence interval (aOR [95% CI]) were most strongly observed for maternal diagnoses of schizophrenia and schizoaffective disorders (2.51 [2.24–2.82]) and depression (2.19 [2.11–2.28]), and paternal diagnoses of schizophrenia and schizoaffective disorders (2.0 [1.75–2.29]) and conduct disorders (1.90 [1.40–2.59]). The aORs for any psychiatric diagnosis were (2.66 [2.54–2.78]) for mothers, (1.95 [1.86–2.04]) for fathers, and (4.50 [4.24–4.79]) for both parents. When both parents had any psychiatric diagnosis, the highest risk was for MD diagnosed at the age of 5–12 years (7.66 [6.60–8.89]); versus at 13–18 years (4.13 [3.85–4.44]) or 19–25 years (3.37 [2.78–4.07]). A stronger association with parental psychopathology and offspring MD was seen among boys than girls, especially among 13–19 years and 19–25 years.

**Conclusions:**

Parental psychiatric disorders, including those diagnosed before offspring birth, were associated with offspring MD, indicating potential genetic and environmental factors in the development of the disorder.

## Introduction

Major depression (MD) is a common mental disorder, with lifetime prevalence estimates of 15–18% (Bromet et al., 2011). It is a major contributor to the global burden of diseases, a leading cause of disability worldwide, and is associated with social and functional impairment (WHO, 2023). The heritability of MD is 40% (Kendler, Ohlsson, Sundquist, & Sundquist, 2018; Sullivan, Neale, & Kendler, 2000). It is strongly postulated that genetic and environmental risk factors interact in the development of this disorder (Penner-Goeke & Binder, 2019).

Several population-based studies have suggested strong familial clustering of MD, and that maternal and paternal depressive symptoms during pregnancy are associated with offspring depressive symptoms later in life (Brennan et al., 2002; Johnson et al., 2006; Kendler, Davis, & Kessler, 1997; Lewis et al., 2017; Marmorstein et al., 2004; Ohannessian et al., 2004). Register-based studies have also shown associations between parental mental disorders and increased risk of offspring depression and other mood disorders (Agerbo et al., 2021; Dean et al., 2010; Li, Sundquist, & Sundquist, 2008; Musliner et al., 2015; Paananen et al., 2021; Thorup et al., 2018). The data based on national registers provide significant advantages while examining these kinds of research questions as it allows the collection of information from a large number of cases, their respective controls, and their parents. However, previous population-based studies have shown offspring MD to associate with the following specific parental psychiatric disorders: schizophrenia, bipolar disorder, MD, obsessive-compulsive disorder, attention deficit-hyperactivity disorder and autism spectrum disorders (Agerbo et al., 2021; Chen et al., 2019; Huang et al., 2021; Musliner et al., 2015; Ostergaard, Waltoft, Mortensen, & Mors, 2013; Taka-Eilola Nèe Riekki, Veijola, Murray, Koskela, & Mäki, 2019; Wang et al., 2022).

Despite the large number of these studies, there are gaps in the research. To our knowledge, no previous population-based study has examined the association between parental psychopathology and offspring MD by dividing the whole spectrum of parental psychopathology into as many different categories as in this study. This provides insights into the specificity of parental transmission of certain mental disorders and the later onset of offspring MD. In addition, many of the previous population-based studies used broad definitions of offspring mood disorders and findings are not specific to MD only. Furthermore, little is known about how parental psychiatric illness influences the vulnerability of MD at different developmental stages in the offspring. Only one previous register-based study has examined parental psychiatric diagnoses and offspring MD across age groups (Musliner et al., 2015), and none has included analyses restricted to parental psychiatric diagnoses prior to offspring birth. Restricting parental diagnosis before birth could prevent bias due to reverse causation – offspring outcomes affecting parental psychiatric diagnosis and mental health help-seeking behavior, which has been rarely addressed by previous studies.

The aim of this study was to examine the association between the whole range of parental psychiatric disorders and offspring MD while controlling for several important demographic and prenatal confounders. In addition, we aimed to examine parental psychiatric diagnoses by the timing of parental diagnoses before their child’s birth and its effect across offspring’s age at first MD diagnosis. We hypothesize that a wide range of parental psychopathology increases the risk of offspring MD and that parental diagnoses before a child’s birth are associated with increased risk for offspring MD. Furthermore, we examined the association between parental psychopathology and offspring MD by the age of onset of MD, which has not been explored in detail previously.

## Methods

### Study design

The present study is a Finnish nationwide population-based study with a nested case–control study design. It utilizes several Finnish national registers were used to collect information on the cases, the controls, and their parents. The source population comprised all singleton children born alive in Finland between January 1987 and December 2007 (1,240,062).

### Information from national registers

Medical diagnoses are routinely registered in the Care Register for Health Care (CRHC), which is maintained by the Finnish Institute of Health and Welfare. Starting from 1969, it includes all diagnoses given in public inpatient care units (psychiatric inpatient treatment in Finland is solely provided by public hospitals). Since 1998, it also includes outpatient diagnoses from public specialized hospital units. In Finland, psychiatric diagnoses are based on the International Classification of Diseases (ICD): ICD-8 from 1969 to 1986, ICD-9 (Finnish version) from 1987 to 1995, and ICD-10 since 1996. The care register was used to identify the cases with MD and it was also the source for both maternal and paternal psychiatric diagnoses. In addition, the personal identity code, date of birth, and sex of the cases were obtained from the care register.

The Digital and Population Data Services Agency (formerly known as the Finnish Population Register Centre) was used to identify the controls, the parents of cases and controls, and to retrieve demographic characteristics of the cases and controls.

The Finnish Medical Birth Register (FMBR), available since 1987, was used to collect information on the child’s birthplace, the mother’s marital status, maternal smoking, maternal socio-economic status (SES), and prenatal growth indicators (gestational age and birthweight).

These national registers were linked together using personal identity codes. Personal identity codes, unique to every individual, were introduced in Finland in the 1960s and are based on date of birth and sex. They are issued to all Finnish citizens and those permanently residing in Finland.

### Cases of major depression

The cases of MD (n = 37,677) included all singleton live-born children in Finland between January 1987 and December 2007 who were diagnosed with depressive episodes or recurrent depression according to the ninth and tenth versions of the International Classification of Diseases (ICD-9 codes [Finnish version] 2961 and ICD-10 codes F32.0-F32.9 and F33.0-F33.9) before December 2015, and after the age of five years. Mild, moderate, and severe depressive episodes and depression with psychotic features were included.

### Controls

The controls (n = 1,45,068) included all singleton live-born children in Finland between January 1987 and December 2007 without any diagnosis of depressive episodes or recurrent depression in the Care Register for Health Care. Controls were matched with cases on date of birth (+/−30 days), sex, place of birth, and residence in Finland at the time of the diagnosis.

We excluded both cases and controls with severe or profound intellectual disability (ICD-9 codes (Finnish version) 3181 and 3182, and ICD-10 codes F72 and F73). These subjects were excluded because of the severity of their condition which might cause difficulty in assessing depression.

### Parental psychiatric diagnoses

The information on parental psychiatric diagnoses was obtained from the Care Register for Health Care. Parents were followed up from January 1969 to December 2015. The parental psychiatric diagnoses according to ICD-10, ICD-9 (Finnish version), and ICD-8 codes, used in the study are listed in Supplementary Table 1 (see Supplementary Table S1, available online).

Parental psychiatric diagnoses were studied in three categories. First, any parental psychiatric diagnoses were aggregated and categorized into a 4-level variable as follows: only mother, only father, both parents, or neither parent having psychiatric diagnoses. Second, maternal and paternal psychiatric disorders were examined separately as present or absent in the following categories, with the indicator variables for each category of disorder simultaneously included in a single model: schizophrenia and schizoaffective disorder; other psychoses; bipolar disorder; unipolar depression and other mood disorders; anxiety disorders; obsessive-compulsive disorder (OCD); eating disorders; personality disorders; alcohol and drug addiction/abuse; attention deficit hyperactivity disorder (ADHD); autism spectrum disorders (ASD); conduct disorders; learning and coordination disorders (LCD); intellectual disability. The parents diagnosed with schizophrenia and other psychotic disorders were not assigned to other groups due to its chronic and pervasive nature. These individuals may be diagnosed with various kinds of psychiatric disorders before the underlying problems (e.g., psychotic illness) become evident. The parents diagnosed with other disorders except these were assigned to several of these groups in case of comorbidity. A similar categorization has been used in other studies (Joelsson et al., 2017; Koskela et al., 2020; Leivonen et al., 2017; Upadhyaya et al., 2019).

Third, any maternal and paternal psychiatric diagnoses were analyzed separately as either as present or absent, based on the timing of the parents’ first diagnosis: before offspring birth versus at any point in time thereafter.

### Covariates

The covariates were included in the analyses based on their potential association with both parental psychopathology and offspring depression as shown in previous literature. They included: maternal and paternal age at birth of the child (Filatova, Upadhyaya, Luntamo, Sourander, & Chudal, 2021), maternal socio-economic status (SES) (Kessler et al., 1997; Sareen, Afifi, McMillan, & Asmundson, 2011), maternal smoking during pregnancy (Mendelsohn, Kirby, & Castle, 2015; Shenassa, 2023), marital status (Su, D’Arcy, & Meng, 2021), parental immigrant status (Straiton, Reneflot, & Diaz, 2014), previous births (Patchen & Lanzi, 2013), and the child’s gestational age (Upadhyaya et al., 2021; Yin et al., 2023), weight for gestational age (Upadhyaya et al., 2021) and region of birth. Region of birth was included because regional disparities in psychiatric diagnoses in Finland have been shown in previous studies. For instance, higher prevalence rates were shown for depression (Filatova et al., 2019) and anxiety (Khanal et al., 2022) in the southern region and higher rates of bipolar disorder (Chudal et al., 2014), and schizophrenia (Suokas et al., 2024) in the eastern part of the country.

Information on maternal age, paternal age, immigrant status, and child’s region of birth was obtained from the Digital and Population Data Services Agency. Maternal smoking, SES, marital status, previous birth, child’s gestational age, and weight for gestational age were derived from the Finnish Medical Birth Register. SES is based on the national Finnish classification of occupations: upper white-collar workers, lower white-collar workers, blue-collar workers, and other workers. Unfortunately, information on paternal SES is not available in the birth register.

Finally, the covariates selected for the adjusted model were based on the covariate testing, which is described in more detail under the subtitle Statistical analyses.

### Validation of major depression diagnosis

We reviewed the medical records of 60 cases of MD registered in the patient record systems of Southwest Finland Hospital District, which provides the secondary and tertiary treatment. The cases selected for the chart review were born between January 1996 and December 2015 and were diagnosed with MD by December 2020. The cases that were diagnosed with MD at or after the age of 5 years, with no comorbid diagnosis of severe or profound intellectual disability or bipolar disorder were included in the review.

Two independent reviewers, both licensed physicians (one specializing in psychiatry and the other being a PhD candidate [MK]), evaluated the medical records for all 60 cases. The review process was supervised by a professor in Child Psychiatry (AS). The medical records were evaluated according to ICD-10 criteria for depression. To determine the final case status, the detailed recordings from the two independent reviewers were compared and reviewed by AS.

For the chart review, medical records of 60 cases of MD (66.67% girls and 33.33% boys) were evaluated from the patient record systems of the Southwest Finland Hospital District. Three cases lacked sufficient information, making chart review evaluation impossible. Of the 57 reviewed cases, two did not meet the diagnostic criteria. The full ICD-10 diagnostic criteria for MD were met in 55 of the 57 cases (96.49%).

### Statistical analyses

We first analyzed the associations between covariates and parental psychopathology among controls and between covariates and offspring MD. Potential covariates were tested using the Pearson’s chi-square test for categorical covariates and t-test for continuous covariates for the association with maternal and paternal psychopathology among controls. Conditional logistic regression models were used to assess the associations between potential covariates and offspring MD. The variables that were associated with parental psychopathology, as well as offspring MD, were selected as covariates in the adjusted model. The limit of significance for selecting covariates into the adjusted model was p < 0.1.

Conditional logistic regression models were used to assess the associations between parental psychiatric diagnoses and offspring outcomes. In the first model, we estimated unadjusted odds ratios (ORs) and two-sided 95% confidence intervals (CIs) for parental psychiatric diagnoses and offspring MD. The associations were also studied for specific paternal and maternal psychiatric diagnoses. In this case, we additionally adjusted for other parent’s psychopathology along with other covariates described above. Moreover, we also examined the association between parental psychiatric diagnoses given before their child’s birth and offspring MD. Additional analyses were conducted for a combined category of parental psychotic or affective disorders before the child’s birth and offspring MD. In the final model, adjustments were made for the covariates mentioned above.

In addition, we used an adjusted conditional logistic regression model including a product term for biological sex X overall parental psychiatric diagnoses category to test for the interaction by sex in the association between parental psychiatric diagnoses and offspring MD. The association between parental psychiatric diagnoses and offspring MD was studied by stratifying by biological sex and by age at diagnosis of MD. The associations were adjusted for the covariates mentioned above. The adjusted association between parental psychiatric diagnoses and offspring MD, by age at diagnosis of MD was visualized as a figure.

A two-sided p-value of <0.05 was interpreted as statistically significant. All statistical analyses were performed with R 4.2.0, Rstudio, version 1.4.1717 (2009–2021 Rstudio, PBC), and data visualization was performed with SAS 9.4.

## Results

The mean age at diagnosis of MD was 16.23 years, with a standard deviation (*SD*) of 3.52 years and a range of 5–25 years. Of all 37,677 cases, 65.52% were girls. 12.43% of cases had both parents diagnosed with any psychiatric disorders, 21.72% had mothers only with any psychiatric disorders, and 15.99% had fathers only with any psychiatric disorders.

The potential confounders that were associated with both maternal and paternal psychiatric diagnoses and MD were: maternal SES, maternal smoking, previous births, maternal marital status, paternal immigrant status, gestational age, weight for gestational age, region of birth, as well as maternal and paternal age at the birth of the child. However, maternal immigrant status was not associated with parental psychopathology in covariate testing. Therefore, all the covariates except maternal immigrant status were included in the adjusted model (see Supplementary Table S2, available online). In addition, the results were adjusted for the other parent’s psychopathology.

Table 1 shows the association between any parental psychiatric diagnoses and offspring MD, overall and also stratified by offspring’s biological sex. Maternal-only psychiatric diagnoses (aOR 2.66, 95% CI 2.54–2.78) and paternal-only psychiatric diagnoses (aOR 1.95, 95% CI 1.86–2.04) were associated with increased odds of offspring MD, but the association was stronger for maternal psychiatric diagnoses when compared to paternal psychiatric diagnoses (p < 0.001). The odds significantly increased when both parents were diagnosed with any psychiatric disorders (aOR 4.50, 95% CI 4.24–4.79) when compared to maternal psychiatric diagnoses (p < 0.001) or paternal psychiatric diagnoses (p < 0.001). There was significant interaction by biological sex in the association between parental psychiatric diagnoses and offspring MD (p < 0.001, df = 3). When the association was stratified by biological sex, increased odds for MD were observed in boys compared to girls in all parental categories.

**Table 1.** Unadjusted and adjusted models, with ORs (95% CI), stratified by offspring sex, for the association between parental psychiatric diagnoses and offspring MD

Parental psychiatric diagnoses	Cases (n = 37,677)	Controls (n = 145,068)	Unadjusted	P-value	Adjusted^a^	P-value
None	18785 (49.86)	105356 (72.63)	Ref		Ref	
Both	4684 (12.43)	5599 (3.86)	4.64 (4.45–4.84)	<0.001	4.50 (4.24–4.79)	<0.001
Only mother	8183 (21.72)	17056 (11.76)	2.67 (2.59–2.76)	<0.001	2.66 (2.54–2.78)	<0.001
Only father	6025 (15.99)	17056 (11.76)	1.97 (1.91–2.04)	<0.001	1.95 (1.86–2.04)	<0.001
Girls	n = 24,688	n = 94,352				
None	12813 (51.90)	68756 (72.87)	Ref		Ref	
Both	2859 (11.58)	3544 (3.76)	4.27 (4.05–4.51)	<0.001	3.79 (3.58–4.01)	<0.001
Only mother	5226 (21.17)	10928 (11.58)	2.54 (2.45–2.64)	<0.001	2.36 (2.27–2.45)	<0.001
Only father	3790 (15.35)	11124 (11.79)	1.82 (1.75–1.90)	<0.001	1.71 (1.64–1.79)	<0.001
Boys	n = 12,989	n = 50,716				
None	5972 (45.98)	36600 (72.17)	Ref		Ref	
Both	1825 (14.05)	2055 (4.05)	5.36 (5.00–5.75)	<0.001	4.68 (4.35–5.05)	<0.001
Only mother	2957 (22.77)	6129(12.08)	2.95 (2.80–3.11)	<0.001	2.72(2.57–2.88)	<0.001
Only father	2235 (17.21)	5932 (11.70)	2.29 (2.17–2.42)	<0.001	2.17 (2.05–2.31)	<0.001

a Adjusted for maternal and paternal age; maternal SES; maternal smoking; marital status; previous births; region of birth; Weight for gestational age; preterm birth and paternal immigration status.

As shown in Table 2, significant associations were observed between all specific psychiatric diagnoses’ categories among mothers and fathers and offspring MD in model 1. When we additionally adjusted for the other parent’s psychiatric diagnoses, and additional psychiatric diagnoses of the same parents, as listed, all categories of parental psychiatric diagnoses were associated with offspring MD except psychoses, OCD, LCD, and intellectual disability in mothers and OCD, eating disorder, ADHD, ASD, LCD and intellectual disability in fathers. In the final model, the highest aORs were observed for maternal diagnoses of schizophrenia and schizoaffective disorders (aOR 2.51, 95% CI 2.24–2.82), unipolar depression (aOR 2.19, 95% CI 2.11–2.28) and ASD (aOR 1.92, 95% CI 1.04–3.56), and paternal diagnoses of schizophrenia and schizoaffective disorders (aOR 2.0, 95% CI 1.75–2.29), conduct disorders (aOR 1.90, 95% CI 1.40–2.59) and unipolar depression (aOR 1.57, 95% CI 1.51–1.65).

**Table 2.** Unadjusted and adjusted models, with ORs (95% CI), for the association between specific parental psychopathology and offspring MD

Parental psychiatric diagnoses		Cases (n = 37,677)	Controls (n = 145,068)	Unadjusted	P-value	^a^Adjusted model 1	P-value	^b^Adjusted model 2	P-value
Schizophrenia and schizoaffective disorder^c^	Maternal	629 (1.67)	899(0.62)	2.71 (2.43–2.99)	<0.001	2.29 (2.05–2.56)	<0.001	2.51 (2.24–2.82)	<0.001
	Paternal	407 (1.10)	698 (0.49)	2.28 (2.01–2.58)	<0.001	2.08 (1.83–2.37)	<0.001	2.0 (1.75–2.29)	<0.001
Other psychoses	Maternal	728 (1.93)	1254 (0.86)	2.26 (2.06–2.48)	<0.001	2.10 (1.91–2.32)	<0.001	1.10 (0.99–1.22)	0.07
	Paternal	659 (1.78)	1220 (0.85)	2.14 (1.94–2.36)	<0.001	1.96 (1.77–2.17)	<0.001	1.16 (1.04–1.29)	0.007
Bipolar disorder	Maternal	1262 (3.35)	1443 (0.99)	3.44 (3.19–3.72)	<0.001	3.03 (2.79–3.29)	<0.001	1.31 (1.20–1.43)	<0.001
	Paternal	834 (2.26)	1398 (0.97)	2.34 (2.14–2.55)	<0.001	2.24 (2.05–2.46)	<0.001	1.20 (1.08–1.32)	<0.001
Unipolar depression and other mood disorders	Maternal	8474 (22.49)	12640 (8.71)	3.03 (2.94–3.13)	<0.001	2.79 (2.70–2.88)	<0.001	2.19 (2.11–2.28)	<0.001
	Paternal	5211 (14.10)	9673 (6.73)	2.28 (2.20–2.37)	<0.001	2.12 (2.04–2.20)	<0.001	1.57 (1.51–1.65)	<0.001
Anxiety disorders	Maternal	2987 (7.93)	4685 (3.23)	2.58 (2.46–2.71)	<0.001	2.30 (2.19–2.42)	<0.001	1.23 (1.16–1.30)	<0.001
	Paternal	1814 (4.91)	3543 (2.47)	2.05 (1.93–2.17)	<0.001	1.83 (1.72–1.95)	<0.001	1.15 (1.07–1.23)	<0.001
- Anxiety disorder without OCD	Maternal	2904 (7.71)	4538 (3.13)	2.59 (2.47–2.72)	<0.001	2.30 (2.19–2.43)	<0.001	1.23 (1.16–1.30)	<0.001
	Paternal	1742 (4.71)	3429 (2.39)	2.03 (1.91–2.15)	<0.001	1.82 (1.71–1.93)	<0.001	1.14 (1.06–1.22)	<0.001
-OCD	Maternal	173 (0.46)	256 (0.18)	2.60 (2.14–3.15)	<0.001	2.60 (2.11–3.19)	<0.001	1.15 (0.92–1.43)	0.20
	Paternal	116 (0.31)	180 (0.13)	2.44 (1.93–3.09)	<0.001	2.34 (1.83–2.99)	<0.001	1.27 (0.98–1.64)	0.06
Eating disorders	Maternal	282 (0.75)	407 (0.28)	2.70 (2.31–3.14)	<0.001	2.37 (2.02–2.79)	<0.001	1.23 (1.03–1.46)	0.01
	Paternal	32 (0.09)	55 (0.04)	2.24 (1.44–3.47)	<0.001	2.07 (1.32–3.27)	<0.001	1.32 (0.82–2.13)	0.24
Personality disorders	Maternal	2313 (6.14)	2354 (1.62)	3.97 (3.74–4.21)	<0.001	3.38 (3.17–3.60)	<0.001	1.44 (1.33–1.55)	<0.001
	Paternal	1999 (5.41)	2880 (2.0)	2.80 (2.64–2.97)	<0.001	2.32 (2.18–2.47)	<0.001	1.33 (1.24–43)	<0.001
Alcohol and drug addiction, abuse	Maternal	3091 (8.20)	4471 (3.08)	2.79 (2.66–2.93)	<0.001	2.19 (2.08–2.30)	<0.001	1.18 (1.12–1.25)	<0.001
	Paternal	5275 (14.28)	10704 (7.45)	2.06 (1.99–2.13)	<0.001	1.80 (1.73–1.87)	<0.001	1.32 (1.26–1.37)	<0.001
ADHD	Maternal	187 (0.50)	193 (0.13)	3.75 (3.06–4.59)	<0.001	2.96 (2.38–3.68)	<0.001	1.36 (1.08–1.71)	<0.001
	Paternal	140 (0.38)	184 (0.13)	2.92 (2.34–3.64)	<0.001	2.35 (1.86–2.98)	<0.001	1.20 (0.93–1.54)	0.14
ASD	Maternal	26 (0.07)	22 (0.02)	4.61 (2.61–8.15)	<0.001	4.26 (2.36–7.67)	<0.001	1.92 (1.04–3.56)	0.03
	Paternal	14 (0.04)	23 (0.02)	2.34 (1.20–4.55)	0.016	2.76 (1.37–5.53)	0.005	1.77 (0.85–3.68)	0.12
Conduct disorders	Maternal	91 (0.24)	87 (0.06)	3.97 (2.96–5.33)	<0.001	2.68 (1.95–3.68)	<0.001	1.91 (1.36–2.69)	<0.001
	Paternal	95 (0.26)	112 (0.08)	3.32 (2.52–4.39)	<0.001	2.60 (1.94–3.47)	<0.001	1.90 (1.40–2.59)	<0.001
LCD	Maternal	75 (0.20)	151 (0.10)	1.90 (1.44–2.51)	<0.001	1.61 (1.19–2.16)	0.002	1.04 (0.76–1.43)	0.79
	Paternal	83 (0.22)	185 (0.13)	1.74 (1.34–2.26)	<0.001	1.55 (1.18–2.04)	0.001	1.07 (0.80–1.42)	0.64
Intellectual disability	Maternal	109 (0.29)	198 (0.14)	2.13 (1.68–2.69)	<0.001	1.82 (1.41–2.35)	<0.001	1.11 (0.84–1.46)	0.43
	Paternal	103 (0.28)	167 (0.12)	2.41 (1.88–3.10)	<0.001	2.02 (1.56–2.62)	<0.001	1.23 (0.94–1.62)	0.12

a Adjusted for maternal and paternal age; maternal SES; maternal smoking; marital status; previous births; region of birth; Weight for gestational age; preterm birth and paternal immigration status.

b Adjusted for all covariates in model 1, additional psychiatric diagnoses of the same parent, as listed and other parent’s psychiatric diagnoses, as listed.

c Hierarchical model: A parent diagnosed with schizophrenia or schizoaffective disorder would only be assigned to this diagnostic category. In other cases, a parent could be assigned to several diagnostic categories.

**Figure 1.**
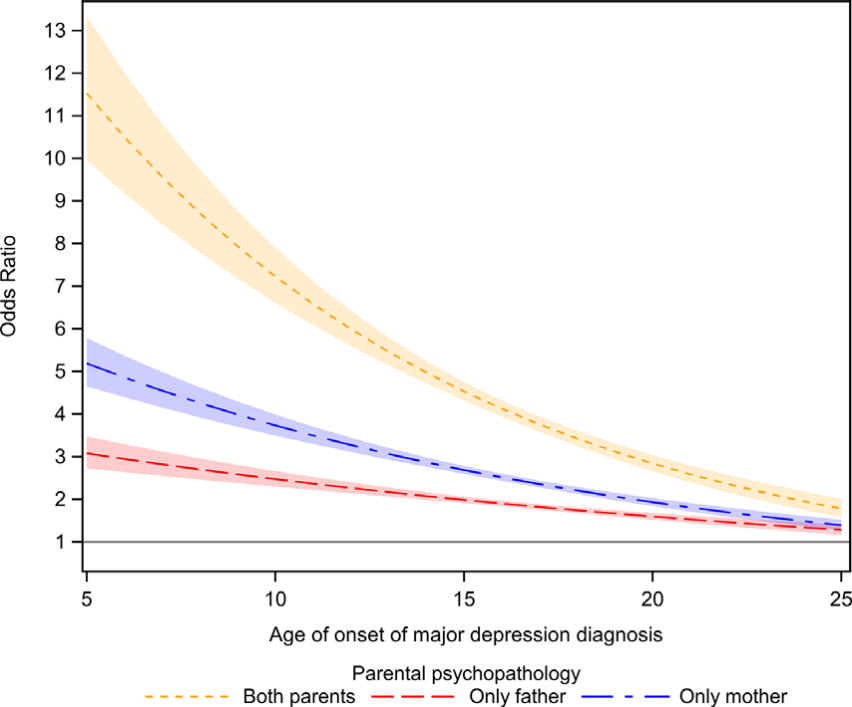
Parental psychopathology and offspring MD, overall by age of onset of offspring MD.

The association between parental psychiatric diagnosis and offspring MD, by age of onset of offspring MD, is shown in Figure 1. The association was adjusted for maternal SES, maternal smoking, previous births, maternal marital status, paternal immigrant status, gestational age, weight for gestational age, region of birth, and maternal and paternal age at the birth of the child.

Table 3 shows the association between parental psychiatric diagnoses and offspring MD by the categories of age at MD diagnosis. When either mother, father, or both had any psychiatric diagnoses, the risk of offspring MD was higher between age 5 and 12 years, than between ages 13 to 18 years or 19 to 25 years. The highest odds for offspring MD were discovered when both parents had any psychiatric diagnosis in the offspring age category 5 to 12 years of age (aOR 7.66, 95% CI 6.60–8.89), then 13 to 18 years of age (aOR 4.13, 95% CI 3.85–4.44) followed by 19 to 25 years of age (aOR 3.37, 95% CI 2.78–4.07). As shown in Supplementary Table S3 (available online), when these associations were stratified by biological sex, the odds for offspring MD were highest for 5 to 12 years of age, then 13 to 18 years of age, or 19 to 25 years of age in both boys and girls.

**Table 3.** Unadjusted and adjusted models, with ORs (95% CI), for the association between parental psychiatric diagnoses and offspring MD by age at onset of MD

Parental psychiatric diagnoses	Cases (n = 37,677)	Controls (n = 145,068)	Unadjusted	P-value	*Adjusted	P-value
5–12 years old	n = 4,490	n = 17,493				
None	1658 (36.93)	12857 (73.50)	Ref		Ref	
Both	842 (18.75)	661 (3.78)	9.68 (8.60–10.90)	<0.001	7.66 (6.60–8.89)	<0.001
Only mother	1309 (29.15)	2076 (11.87)	4.84 (4.43–5.30)	<0.001	4.23 (3.79–4.72)	<0.001
Only father	681 (15.17)	1899 (10.86)	2.77 (2.50–3.08)	<0.001	2.50 (2.20–2.84)	<0.001
13–18 years old	n = 23,552	n = 90,513				
None	11810 (50.14)	65854 (72.76)	Ref		Ref	
Both	2883 (12.24)	3463 (3.83)	4.60 (4.36–4.86)	<0.001	4.13 (3.85–4.44)	<0.001
Only mother	5031 (21.36)	10666 (11.78)	2.62 (2.52–2.72)	<0.001	2.46 (2.33–2.59)	<0.001
Only father	3828 (16.25)	10530 (11.63)	2.02 (1.94–2.11)	<0.001	1.92 (1.81–2.03)	<0.001
19–25 years old	n = 9,635	n = 37,062				
None	5317 (55.18)	26645 (71.89)	Ref		Ref	
Both	959 (9.95)	1475 (3.98)	3.22 (2.95–3.52)	<0.001	3.37 (2.78–4.07)	<0.001
Only mother	1843 (19.13)	4315 (11.64)	2.12 (1.99–2.26)	<0.001	2.23 (1.95–2.55)	<0.001
Only father	1516 (15.73)	4627 (12.48)	1.63 (1.53–1.74)	<0.001	1.60 (1.39–1.84)	<0.001

* Adjusted for maternal and paternal age; maternal SES; maternal smoking; marital status; previous births; region of birth; Weight for gestational age; preterm birth and paternal immigration status.

Additionally, as shown in Table 4, we examined the association between parental psychiatric diagnoses given before their child’s birth and offspring MD. Maternal psychiatric diagnoses (aOR 2.31, 95% CI 2.09–2.55) and paternal psychiatric diagnoses (aOR 1.89, 95% CI 1.76–2.03) before their child’s birth were associated with increased odds of offspring MD. The odds significantly increased when both parents were diagnosed with any psychiatric disorder (aOR 3.50, 95% CI 2.80–4.38). When the associations were stratified by biological sex, increased odds for MD were observed in boys compared to girls in all parental categories (Table 4).

**Table 4.** Unadjusted and adjusted models, with ORs (95% CI), for the association between parental psychiatric diagnoses before the child’s birth and offspring MD, overall and by offspring sex

Parental psychiatric diagnoses	Cases (n = 37,677)	Controls (n = 145,068)	Unadjusted	P-value	Adjusted^a^	P-value
None	33664 (89.35)	137943 (95.09)	Ref		Ref	
Both	339 (0.90)	287 (0.20)	4.84 (4.13–5.67)	<0.001	3.50 (2.80–4.38)	<0.001
Only mother	1280 (3.40)	2145 (1.48)	2.46 (2.30–2.64)	<0.001	2.31 (2.09–2.55)	<0.001
Only father	2394 (6.35)	4693 (3.24)	2.10 (2.30–2.64)	<0.001	1.89 (1.76–2.03)	<0.001
Girls						
None	22255 (90.15)	89812 (95.19)	Ref		Ref	
Both	195 (0.79)	194 (0.21)	4.05 (3.31–4.95)	<0.001	3.07 (2.48–3.79)	<0.001
Only mother	770 (3.12)	1329 (1.41)	2.36 (2.15–2.58)	<0.001	2.06 (1.87–2.27)	<0.001
Only father	1468 (5.95)	3017 (3.20)	1.97 (1.84–2.10)	<0.001	1.72 (1.61–1.85)	<0.001
Boys						
None	11409 (87.84)	48131 (94.90)	Ref		Ref	
Both	144 (1.11)	93 (0.18)	6.52 (5.02–8.47)	<0.001	4.88 (3.71–6.42)	<0.001
Only mother	510 (3.93)	816 (1.61)	2.65 (2.36–2.97)	<0.001	2.23 (1.97–2.52)	<0.001
Only father	926 (7.13)	1676 (3.30)	2.35 (2.17–2.56)	<0.001	2.01 (1.84–2.20)	<0.001

a Adjusted for maternal and paternal age; maternal SES; maternal smoking; marital status; previous births; region of birth; Weight for gestational age; preterm birth and paternal immigration status.

Supplementary Table S4 (available online) shows the association between parental psychiatric diagnoses given before their child’s birth and offspring MD stratified by age at MD diagnosis. Parental psychiatric diagnoses before a child’s birth were significantly associated with offspring MD in all age groups when both parents only the mother, or only the father was diagnosed. Odds ratios were highest in the age group first diagnosed at 5–12 years, compared to age groups 13–18 years and 19–25 years. In all groups, the odds were highest when both parents were diagnosed with any psychiatric disorder before the child’s birth.

Tables S5 and S6 (available online) show the additional analyses for parental psychotic and/or affective diagnosis before the child’s birth and offspring MD. Affective and/or psychotic disorder diagnoses prior to the child’s birth were associated with offspring MD when both parents (aOR 3.06, 95% CI 1.67–5.52), when only the mother (aOR 2.36, 95% CI 2.03–2.74) and when only father (aOR 1.94, 95% CI 1.65–2.29) were diagnosed. When the association was stratified by age of MD diagnosis, increased odds of MD were observed in all age groups.

## Discussion

The present study investigated a wide range of diagnostic categories of parental psychiatric disorders and offspring MD, examining the risk by sex, and age at first MD diagnosis, and controlling for several potential confounders, including either parent’s psychiatric diagnoses that account for effects related to assortative mating. The study had several findings: (1) most of the examined categories of parental psychopathology were associated with offspring MD, both maternal and paternal psychiatric diagnoses were associated with offspring MD, and stronger associations were seen when both parents had any psychiatric diagnosis, (2) maternal psychiatric diagnoses had stronger impacts than paternal psychiatric diagnoses, (3) significant interaction by biological sex was observed when either or both parents had any psychiatric diagnosis, with higher odds for boys, and (4) parental psychopathology was associated more strongly with earlier onset MD.

The present study shows offspring MD to be associated with an even wider range of parental psychiatric disorders than previously known. Several of the previous register-based studies have examined the association between many types of parental psychopathology and offspring mood disorders, but not offspring MD specifically (Dean et al., 2010; Gottesman, Laursen, Bertelsen, & Mortensen, 2010; Li et al., 2008; Paananen et al., 2021; Thorup et al., 2018). However, the association between parental MD (Klein, Lewinsohn, Seeley, & Rohde, 2001; Ohannessian et al., 2004), ADHD (Chen et al., 2019), OCD (Huang et al., 2021), bipolar disorder (Chen et al., 2019), schizophrenia (Cheng et al., 2018), ASD (Wang et al., 2022), and offspring MD have been reported in previous population-based studies. A register-based study from Finland also examined parental psychiatric hospitalization for several conditions and the risk of offspring mood disorders, but only parental psychiatric hospitalization after a child’s birth was considered (Paananen et al., 2021). The emerging literature has strongly suggested that the offspring of parents with severe mental illness are at increased risk of mental disorders. A meta-analysis of Family High-risk studies reported limited specificity of mental disorders, and overlap of familial and genetic risk to various mental disorders (Rasic et al., 2014). The risk extending across diagnostic boundaries, or the heterotypic transmission suggests shared genetic factors underlying schizophrenia, bipolar disorder, and major depression (Cross-Disorder Group of the Psychiatric Genomics Consortium, 2013).

In this study, offspring MD was more strongly associated with maternal psychiatric diagnoses than paternal psychiatric diagnoses. This applied to both overall diagnostic categories (any parental psychiatric diagnosis) as well as specific psychiatric diagnostic categories, including depression, bipolar disorder, anxiety, and personality disorder but not alcohol drug addiction, and abuse. Previous studies have well-established associations between several maternal psychiatric diagnoses and offspring MD, but the results of the associations between paternal-specific psychiatric disorders and offspring MD are more limited. It is likely that mothers with psychiatric illnesses are less responsive to an infant’s needs, which could affect their caregiving behavior (Priel, Zeev-Wolf, Djalovski, & Feldman, 2020). Moreover, several prenatal factors, such as substance use, smoking, obstetric complications, maternal stress during pregnancy, or maternal medication use perinatally could also function as risk factors for offspring depression (Malm et al., 2016; Serati et al., 2020; Shenassa et al., 2023). Although, we controlled for several maternal factors in the present study, the possibility of residual confounding remains.

When the data was stratified by biological sex, a stronger association with parental psychopathology and offspring MD was seen among boys than girls, especially in the age group 13–19 and 19–25 years old. This suggests that some form of depression that is not related to parental psychopathology arises among females during adolescence and may play a role in the development of a gender difference in MD prevalence during adolescence. The findings of previous studies are inconsistent, with some, but not all, being in line with the results of this study. Our finding is consistent with a Danish register study, in which the authors speculated that the finding could be explained by increased help-seeking among depressed males having a family member with a previous mental disorder (Musliner et al., 2015). Moreover, in Finland, although girls were significantly more likely than boys to use specialized psychiatric outpatient care and receive mental disorder diagnoses, an age-specific pattern by sex was observed; the median age for the first instance of specialized psychiatric inpatient care was 12 years for boys and 15 years for girls (Paananen et al., 2013). However, a Swedish register-based study found no significant differences in familial risks of mood disorders between the sexes, including transmission from father and mother, separately (Li et al., 2008).

When we restricted parental psychiatric diagnoses to those before the offspring’s birth, the associations remained significant. The benefit of restricting to this period is that the direction of possible causality is known, since the associations must be due to child-independent factors, such as genetic predisposition. Conversely, when examining parental psychopathology at any point in time, the associations may partly be due to reverse causation (offspring depression influencing the risk of a diagnosis of psychiatric disorders in parents). Significant associations were also observed for parental psychotic and affective disorders before offspring birth and offspring MD. Restricting to more severe disorders such as parental psychotic and/or affective disorders guarantees that the associations are not impacted by less severe/shorter/reactive disorders, but rather by more serious and long-lasting parental psychiatric disorders. Previous studies have strongly suggested that parental depression is associated with offspring depression during childhood, adolescence, and adulthood (Rajyaguru, Kwong, Braithwaite, & Pearson, 2021). However, there are limited studies that have examined other parental psychiatric diagnoses before or during a child’s birth and subsequent risk of offspring depression. Previous register-based studies from Denmark (Musliner et al., 2015), Sweden (Li et al., 2008) and Finland (Paananen et al., 2021) did not restrict parental psychiatric disorders to those occurring before their child’s birth, and only parental psychiatric hospitalization after the child’s birth was considered in the Swedish and Finnish studies.

Regarding psychiatric disorders having a genetic overlap with MD, the evidence is strongest for different types of affective and psychotic disorders (Demirkan et al., 2011; Kessler, Merikangas, & Wang, 2007; Sullivan et al., 2000). It is noteworthy, that while in our study parental psychotic and affective disorders diagnosed before offspring birth were associated with offspring MD as expected, the strength of the associations did not stand out compared to analyses conducted on different types of parental psychiatric disorders diagnosed at any point in time. Moreover, most types of parental psychiatric disorders diagnosed at any point in time were associated with offspring MD.

The association between offspring MD and any parental psychopathology (diagnosis given at any point of time) in our study was markedly elevated in the case of two affected parents, particularly in the youngest age group (offspring being diagnosed with MD at the age of 5–12 years of age). The respective association remained significant when restricting to parental diagnoses given prior to offspring birth. This finding of our study is consistent with previous Danish and Swedish register-based studies (Li et al., 2008; Musliner et al., 2015). The Danish register-based study (Musliner et al., 2015) reported the strongest effect of maternal and paternal affective disorders (unipolar or bipolar) in the youngest age groups, then the risk decreased steadily with offspring age. However, the effect of maternal and paternal anxiety remained relatively stable over the offspring ages and the effect of paternal schizophrenia increased over time while the effect of maternal schizophrenia remained stable (Musliner et al., 2015). Similarly, a Swedish register-based study that investigated the risk for mood disorders at different age groups among children of parents with mood disorders found that the risk was highest in the youngest age groups, and remained stable after 30 years of age (Li et al., 2008). The association between parental psychiatric diagnoses before the child’s birth and offspring depression at an early age may be explained by genetic vulnerability to depression. A recent study suggested that parental history along with polygenic liability was associated with an elevated risk of early-onset depression (Agerbo et al., 2021). While genetic factors may explain the association, brain development is highly sensitive to environmental influences. Early life exposure to both genetic and environmental factors could increase offspring’s vulnerability to the development of early psychopathology. For instance, parental psychiatric disorders could influence the attachment between the parent and the child, which is an important risk factor for several psychiatric disorders (Atkinson et al., 2000; Upadhyaya et al., 2019). Parents with severe psychiatric disorders may not be readily available for their children. Moreover, studies suggest that parenting styles also influence the development of depression in children (Eun, Paksarian, He, & Merikangas, 2018).

The limitations of the present study should also be considered while interpreting the findings. First, the MD diagnoses were derived from the Care Register for Health Care, which included diagnoses from public specialized mental health services. This may have missed milder cases of MD diagnosed in primary care settings or private clinics. Therefore, this study may to some degree underestimate the strength of the associations. Moreover, our study sample was restricted to those whose psychiatric disorders have been recognized and diagnosed in Finnish registers. It is likely that parents with certain psychiatric diagnoses may not seek treatment and therefore do not end up in the register. However, this would be similar for the parents of both cases and controls. Second, the diagnoses were based on clinical evaluations or diagnostic interviews by psychiatrists rather than standardized interviews. However, chart review validation showed that 96.49% of cases met the full ICD-10 diagnosis criteria for MD. Finally, the limited age range of 5 to 25 years may have skewed the case mix to early onset MD, but it should also be noted that the number of cases in each group of age of onset of MD was adequate to maintain statistical power.

## Conclusion

The children of parents with psychiatric disorders were at increased risk for major depression. The risk was particularly high when both parents were diagnosed with psychiatric disorders. Parental psychiatric disorders, including those diagnosed before the child’s birth, were strongly associated with offspring MD, indicating potential genetic and environmental interactions in the development of the disorder. The risk was markedly elevated in the case of two affected parents, to a greater extent in the youngest age group (5–12 years) than in adolescence (13–18 years) or in young adulthood (19–25 years).

## Supporting information

Upadhyaya et al. supplementary material Upadhyaya et al. supplementary material

## Supplementary material

The supplementary material for this article can be found at http://doi.org/10.1017/S0033291725000662.
