# Supplementary material for: Parental psychiatric disorders and age-specific risk for offspring major depression: Finnish nationwide register-based study
Source: Psychol Med. 2025 Mar 26;55:e96. doi: 10.1017/S0033291725000662 (PMC12094644; doi:10.1017/S0033291725000662)

Table S1: The diagnostic categories for parental psychopathology

|  | **ICD-10** | **ICD-9 (Finnish Version)** | **ICD-8** |
| --- | --- | --- | --- |
| **Schizophrenia and schizoaffective disorder** | F20, F25 | 295 | 295 |
| **Psychoses, other than schizophrenia and schizoaffective disorder** | F21-24, F28, F29 | 297, 2988A, 2989X, 3012C | 297, 298.20, 298.30, 298.99, 299 |
| **Bipolar disorder** | F30, F31 | 2962A-G, 2963A-G, 2964A-G, 2967A | 296.10, 296.30, 298.10 |
| **Unipolar depression and other mood disorders** | F32, F33, F34, F38, F39 | 2961A-G, 2968A, 3004A | 296 (excluding 296.10 and 296.30), 298.00, 300.41 |
| **Anxiety disorders** | F40-42 (excluding F41.2) | 3000A, 3000B, 3000C, 3002B, 3002C, 3002D, 3002X, 3003A | 300.00, 300.20, 300.30 |
| **Sub-analysis:** |  |  |  |
| **Anxiety disorders, other than OCD** | F40-41 (excluding F41.2) | 3000A, 3000B, 3000C, 3002B, 3002C, 3002D, 3002X | 300.00, 300.20 |
| **OCD** | F42 | 3003A | 300.30 |
| **Eating disorders** | F50 | 3071A, 3075A, 3075B, 3075C, 3075E | 306.50 |
| **Personality disorders** | F60-62 | 301 (excluding 3012C) | 301 |
| **Alcohol and drug addiction/abuse** | F10-19 | 303–305, 291–292 | 303–304, 291, 294.30 |
| **ADHD** | F90 | 314 |  |
| **Autism** | F84 | 299 |  |
| **Conduct disorders** | F91, F92 | 3120A, 3123D | 308.99 |
| **Learning and coordination disorders** | F80-83 | 315 | 306.00, 306.10, 306.30 |
| **Intellectual disability** | F70–F79 | 317-319 | 310-315 |
| **Other psychiatric disorders** | F41.2, F43-45, F48, F51-55, F59, F63-66, F68-69, F88-89, F93-95(excluding F95.2), F99 | 300 (excluding 3000A, 3000B, 3000C, 3002B, 3002C, 3002D, 3002X, 3003A, 3004A and 3012C), 302, 3074A, 3074F, 3074H, 3078A, 3079X, 309 (excluding 3092A, 3092B, 3093A and 3094A), 312 (excluding 3120A and 3123D) | 300(excluding 300.00, 300.20, 300.30, 300.41), 302 (excluding 302.00), 305, 306.20, 306.40, 306.98, 307.99, |

Table S2. Covariates in relation to parental psychopathology among controls and in relation to risk of offspring MD

|  | **Maternal psychopathology** | | | **Paternal psychopathology** | | | **Relation between covariates and offspring MD** |
| --- | --- | --- | --- | --- | --- | --- | --- |
| **Covariates** | **Yes** | **No** | **P-value** | **Yes** | **No** | **P-value** | **P-value** |
| **Maternal socioeconomic status** |  |  | <0.001 |  |  | <0.001 | <0.001 |
| Upper white-collar worker | 1390 (6.14) | 11055 (9.03) |  | 1282 (5.66) | 11163 (9.12) |  |  |
| Lower white-collar worker | 5535 (24.43) | 34840 (28.46) |  | 5591 (24.68) | 34784 (28.42) |  |  |
| Blue-collar worker | 3057 (13.49) | 13287 (10.85) |  | 3141 (13.86) | 13203 (10.79) |  |  |
| Others | 2527 (11.15) | 10273 (8.396) |  | 2158 (9.53) | 10642 (8.69) |  |  |
| Missing | 10147(44.79) | 52957 (43.26) |  | 10483 (46.27) | 52621 (42.99) |  |  |
| **Maternal smoking^1^** |  |  | <0.001 |  |  | <0.001 | <0.001 |
| Yes | 5430 (24.70) | 15540 (13.09) |  | 5375 (24.39) | 15595 (13.14) |  |  |
| No | 16550 (75.30) | 103180 (86.91) |  | 16664(75.61) | 103066 (86.86) |  |  |
| **Previous births^2^** |  |  | <0.001 |  |  | <0.001 | 0.02 |
| None | 6330 (28.21) | 37886 (31.27) |  | 6602 (29.39) | 37614 (31.05) |  |  |
| Yes | 16108 (71.79) | 83283 (68.73) |  | 15861 (70.61) | 83530 (68.95) |  |  |
| **Marital status** |  |  | <0.001 |  |  | <0.001 | <0.001 |
| Married/in a relationship | 20070 (88.59) | 112944 (92.27) |  | 20117 (88.80) | 112897 (92.23) |  |  |
| Single | 406 (1.79) | 1275 (1.04) |  | 368 (1.62) | 1313 (1.07) |  |  |
| Unknown | 2180 (9.62) | 8193 (6.69) |  | 2170 (9.58) | 8203 (6.70) |  |  |
| **Paternal immigrant status^3^** |  |  |  |  |  | 0.001 | <0.001 |
| Immigrated | 457 (2.05) | 2178 (1.79) | 0.009 | 356 (1.57) | 2279 (1.88) |  |  |
| Not immigrated | 21833 (97.95) | 119175 (98.21) |  | 22299 (98.43) | 118709 (98.12) |  |  |
| **Maternal immigrantstatus^4^** |  |  | 0.77 |  |  | 0.49 | 0.004 |
| Immigrated | 318 (1.40) | 1748 (1.43) |  | 334 (1.47) | 1732 (1.42) |  |  |
| Not immigrated | 22338 (98.60) | 120652 (98.57) |  | 22320 (98.53) | 120670 (98.58) |  |  |
| **Weight for gestational age^5^** |  |  | <0.001 |  |  | <0.001 | <0.001 |
| <-2SD | 761 (3.40) | 3288 (2.72) |  | 759 (3.39) | 3290 (2.72) |  |  |
| -2SD to +2 SD | 20681 (92.42) | 113026(93.58) |  | 20845 (93.09) | 112862 (93.46) |  |  |
| >+2SD | 935 (4.18) | 4467 (3.70) |  | 789 (3.52) | 4613 (3.82) |  |  |
| **Gestational age (weeks)^6^** |  |  | <0.001 |  |  | 0.001 | <0.001 |
| <37 | 1378 (6.08) | 6159 (5.03) |  | 1282 (5.66) | 6255 (5.10) |  |  |
| 37-41 | 20271 (89.39) | 110958 (90.56) |  | 20356 (89.79) | 110873 (90.49) |  |  |
| >=42 | 1027 (4.53) | 5404 (4.41) |  | 1032 (4.55) | 5399 (4.41) |  |  |
| **Region of birth^7^** |  |  | <0.001 |  |  | <0.001 | <0.001 |
| Eastern | 2734 (12.07) | 13336 (10.89) |  | 2781 (12.28) | 13279 (10.85) |  |  |
| Northern | 3033 (13.39) | 18287 (14.94) |  | 3080 (13.60) | 18240 (14.90) |  |  |
| Southern | 9098 (40.16) | 47498 (38.80) |  | 9096 (40.15) | 47500 (38.80) |  |  |
| Western | 7791 (34.39) | 43301 (35.37) |  | 7698 (33.98) | 43394 (35.45) |  |  |
| **Maternal age^8^** | Mean | |  | Mean | |  |  |
|  | 28.35 | 28.89 | <0.001 | 20.18 | 20.93 | <0.001 | <0.001 |
| **Paternal age^9^** |  |  |  |  |  |  |  |
|  | 30.95 | 31.20 | <0.001 | 30.87 | 31.22 | <0.001 | <0.001 |

^1^ Data missing for 1209 cases and 4368 controls; ^2^ Data missing for 437 cases and 1461 controls; ^3^ Data missing for 730 cases and 1425 controls; ^4^ Data missing for 1 case and 12 controls; ^5^ Data missing for 568 case and 1910 controls; ^6^ Data missing for 556 cases and 1870 controls; ^7^Data missing for 1 case; ^8^ Data missing for 1 case and 12 controls; ^9^Data missing for 730 cases and 1425 controls

Table S3. Unadjusted and adjusted models, with ORs (95% CI), for the association between parental psychiatric diagnoses and offspring MD by age at onset of MD and biological sex

| **Parental**  **psychiatric diagnoses** | **Cases**  **n=24,688** | **Controls**  **n=94,352** | **Unadjusted** | **Adjusted^1^** | **Cases**  **n=12,989** | **Controls**  **n=50,716** | **Unadjusted** | **Adjusted^1^** |
| --- | --- | --- | --- | --- | --- | --- | --- | --- |
|  | **Girls** | | | | **Boys** | | | |
| **5-12**  **years old** | **n=1,512** | **n=5,786** |  |  | **n=2978** | **n=11,707** |  |  |
| None | 564 (37.30) | 4253 (73.51) | Ref | Ref | 1094 (36.74) | 8604 (73.49) | Ref | Ref |
| Both | 281 (18.58) | 204 (3.53) | 9.95 (8.07-12.26)* | 8.42 (6.73-10.54)* | 561 (18.84) | 457 (3.90) | 9.53 (8.25-11.01)* | 7.80 (6.67-8.12)* |
| Only mother | 465 (30.75) | 682 (11.79) | 5.04 (4.33-5.87)* | 4.45 (3.77-5.26)* | 844 (28.34) | 1394 (11.91) | 4.73 (4.24-5.28)* | 4.20 (3.73-4.73)* |
| Only father | 202 (13.36) | 647 (11.18) | 2.41 (2.00-2.90)* | 2.22 (1.82-2.71)* | 479 (16.08) | 1252 (10.69) | 2.97 (2.61-3.36)* | 2.67 (2.33-3.05)* |
| **13-18**  **years old** | **n=17,267** | **n=66,007** |  |  | **n=6,285** | **n=24,506** |  |  |
| None | 8885 (51.46) | 48238 (73.08) | Ref | Ref | 2925 (46.54) | 17616 (71.88) | Ref | Ref |
| Both | 2009 (11.63) | 2467 (3.74) | 4.37 (4.10-4.66)* | 3.86 (3.61-4.13)* | 874 (13.91) | 996 (4.06) | 5.22 (4.72-5.77)* | 4.78 (4.30-5.32)* |
| Only mother | 3661 (21.20) | 7652 (11.59) | 2.58 (2.46-2.70)* | 2.39 (2.28-2.51)* | 1370 (21.80) | 3014 (12.30) | 2.74 (2.54-2.95)* | 2.55 (2.36-2.77)* |
| Only father | 2712 (21.20) | 7652 (11.59) | 1.92 (1.82-2.02)* | 1.78 (1.69-1.88)* | 1116 (17.76) | 2880 (11.75) | 2.33 (2.15-2.52)* | 2.23 (2.05-2.42)* |
| **19-25**  **years old** | **n=5,909** | **n=22,559** |  |  | **n=3,726** | **n=14,503** |  |  |
| None | 3364 (56.93) | 16265 (72.10) | Ref | Ref | 1953 (52.42) | 10380 (71.57) | Ref | Ref |
| Both | 569 (9.63) | 873 (3.87) | 3.12 (2.79-3.49)* | 2.88 (2.55-3.24)* | 390 (10.47) | 602 (4.15) | 3.38 (2.95-3.88) | 2.93 (2.53-3.39)* |
| Only mother | 1100 (18.62) | 2594 (11.50) | 2.03 (1.87-2.19)* | 1.92 (1.77-2.09)* | 743 (19.94) | 1721 (11.87) | 2.29 (2.07-2.52) | 2.18 (1.96-2.42)* |
| Only father | 876 (14.82) | 2827 (12.53) | 1.48 (1.37-1.62)* | 1.47 (1.35-1.60)* | 640 (17.18) | 1800 (12.41) | 1.87 (1.69-2.08) | 1.83 (1.64-2.03)* |

*P<0.001; ^1^Adjusted for maternal and paternal age; maternal SES; maternal smoking; marital status; previous births; region of birth; Weight for gestational age; preterm birth and paternal immigration status

Table S4. Unadjusted and adjusted models, with ORs (95% CI), for the association between parental psychiatric diagnoses before child’s birth and offspring MD by age at onset of MD.

| **Parental**  **psychiatric diagnoses** | **Cases (n=37,677)** | **Controls (n=145,068)** | **Unadjusted** | **P-value** | **Adjusted^1^** | **P-value** |
| --- | --- | --- | --- | --- | --- | --- |
| **5-12 years old** | **n=4,490** | **n=17,493** |  |  |  |  |
| None | 3689 (82.16) | 16469 (94.15) | Ref |  | Ref |  |
| Both | 86 (1.92) | 50 (0.29) | 7.68 (5.39-10.93) | <0.001 | 4.77 (3.08-7.37) | <0.001 |
| Only mother | 289 (6.44) | 336 (1.92) | 3.93 (3.34-4.63) | <0.001 | 3.20 (2.60-3.94) | <0.001 |
| Only father | 426 (9.49) | 638 (3.65) | 3.01 (2.65-3.43) | <0.001 | 2.35 (2.00-2.76) | <0.001 |
| **13-18 years old** | **n=23,552** | **n=90,513** |  |  |  |  |
| None | 21123 (89.69) | 86160 (95.19) | Ref |  | Ref |  |
| Both | 180 (0.76) | 168 (0.19) | 4.36 (3.53-5.39) | <0.001 | 3.29 (2.48-4.36) | <0.001 |
| Only mother | 753 (3.20) | 1274 (1.41) | 2.43 (2.22-2.66) | <0.001 | 2.13 (1.88-2.41) | <0.001 |
| Only father | 1496 (6.35) | 2914 (3.22) | 2.10 (1.97-2.24) | <0.001 | 1.83 (1.69-1.99) | <0.001 |
| **19-25 years old** | **n=9,635** | **n=37,062** |  |  |  |  |
| None | 8852 (91.87) | 35314 (95.28) | Ref |  | Ref |  |
| Both | 73 (0.76) | 69 (0.19) | 4.20 (3.02-5.85) | <0.001 | 2.10 (1.002-4.43) | <0.001 |
| Only mother | 238 (2.47) | 538 (1.45) | 1.76 (1.51-2.06) | <0.001 | 1.71 (1.22-1.41) | <0.001 |
| Only father | 472 (4.90) | 1141 (3.08) | 1.65 (1.48-1.85) | <0.001 | 1.54 (1.22-1.93) | <0.001 |

^1^Adjusted for maternal and paternal age; maternal SES; maternal smoking; marital status; previous births; region of birth; Weight for gestational age; preterm birth and paternal immigration status

Table S5. Unadjusted and adjusted models, with ORs (95% CI), for the association between parental psychotic and or affective diagnosis before child’s birth and offspring MD

| **Parental psychotic and/or affective diagnoses^a^** | **Cases (n=37,677)** | **Controls (n=145,068)** | **Unadjusted** | **P-value** | **Adjusted^1^** | **P-value** |
| --- | --- | --- | --- | --- | --- | --- |
| None | 37052 (98.34) | 144046 (99.30) | Ref |  | Ref |  |
| Both | 25 (0.07) | 29 (0.02) | 3.43 (2.01-5.86) | <0.001 | 3.06 (1.67-5.52) | <0.001 |
| Only mother | 349 (0.93) | 511 (0.35) | 2.65 (2.31-3.04) | <0.001 | 2.36 (2.03-2.74) | <0.001 |
| Only father | 251 (0.67) | 482 (0.33) | 2.03 (1.74-2.37) | <0.001 | 1.94 (1.65-2.29) | <0.001 |

^1^Adjusted for maternal and paternal age; maternal SES; maternal smoking; marital status; previous births; region of birth; Weight for gestational age; preterm birth and paternal immigration status

^a^ICD-10 codes: F20-29 and F30-39; ICD 9 codes: 295 297, 2988A, 2989X, 3012C, 2962A-G, 2963A-G, 2964A-G, 2967A, 2961A-G, 2968A, 3004A; ICD 8 codes: 295, 297, 298.20, 298.30, 298.99, 299, 296.10, 296.30, 298.10, 296 (excluding 296.10 and 296.30), 298.00, 300.41

Table S6. Unadjusted and adjusted models, with ORs (95% CI), for the association between parental psychotic and or affective diagnosis before child’s birth and offspring MD by age at onset of MD

| **Parental psychotic and/or affective diagnoses^a^** | **Cases (n=37,677)** | **Controls (n=145,068)** | **Unadjusted** | **P-value** | **Adjusted^1^** | **P-value** |
| --- | --- | --- | --- | --- | --- | --- |
| **5-12**  **years old** | **n=4,490** | **n=17,493** |  |  |  |  |
| None | 4323 (96.28) | 17298 (98.89) | Ref |  | Ref |  |
| Both | 9 (0.20) | 6 (0.03) | 6.20 (2.20-17.46) | <0.001 | 3.89 (1.21-12.46) | 0.02 |
| Only mother | 99 (2.20) | 114 (0.65) | 3.48 (2.65-4.57) | <0.001 | 2.94 (2.29-3.97) | <0.001 |
| Only father | 59 (1.31) | 75 (0.43) | 3.19 (2.26-4.51) | <0.001 | 3.13 (2.15-4.57) | <0.001 |
| **13-18**  **years old** | **n=23,552** | **n=90,513** |  |  |  |  |
| None | 23226 (98.62) | 89926 (99.35) | Ref |  | Ref |  |
| Both | 10 (0.04) | 18 (0.02) | 2.22 (1.02-4.81) | 0.04 | 2.30 (0.96-5.47) | 0.06 |
| Only mother | 180 (0.76) | 283 (0.31) | 2.46 (2.04-2.97) | <0.001 | 2.19 (1.78-2.69) | <0.001 |
| Only father | 136 (0.58) | 286 (0.32) | 1.85 (1.50-2.27) | <0.001 | 1.75 (1.41-2.17) | <0.001 |
| **19-25**  **years old** | **n=9,635** | **n=37,062** |  |  |  |  |
| None | 9503 (98.63) | 36822 (99.35) | Ref |  | Ref |  |
| Both | 6 (0.06) | 5 (0.01) | 4.51 (1.37-14.84) | 0.01 | 3.83 (1.13-12.92) | 0.03 |
| Only mother | 70 (0.73) | 114 (0.31) | 2.33 (1.73-3.15) | <0.001 | 2.13 (1.54-2.93) | <0.001 |
| Only father | 56 (0.58) | 121 (0.33) | 1.79 (1.30-2.46) | <0.001 | 1.76 (1.26-2.46) | <0.001 |

^1^Adjusted for maternal and paternal age; maternal SES; maternal smoking; marital status; previous births; region of birth; Weight for gestational age; preterm birth and paternal immigration status

^a^ICD-10 codes: F20-29 and F30-39; ICD 9 codes: 295 297, 2988A, 2989X, 3012C, 2962A-G, 2963A-G, 2964A-G, 2967A, 2961A-G, 2968A, 3004A; ICD 8 codes: 295, 297, 298.20, 298.30, 298.99, 299, 296.10, 296.30, 298.10, 296 (excluding 296.10 and 296.30), 298.00, 300.41


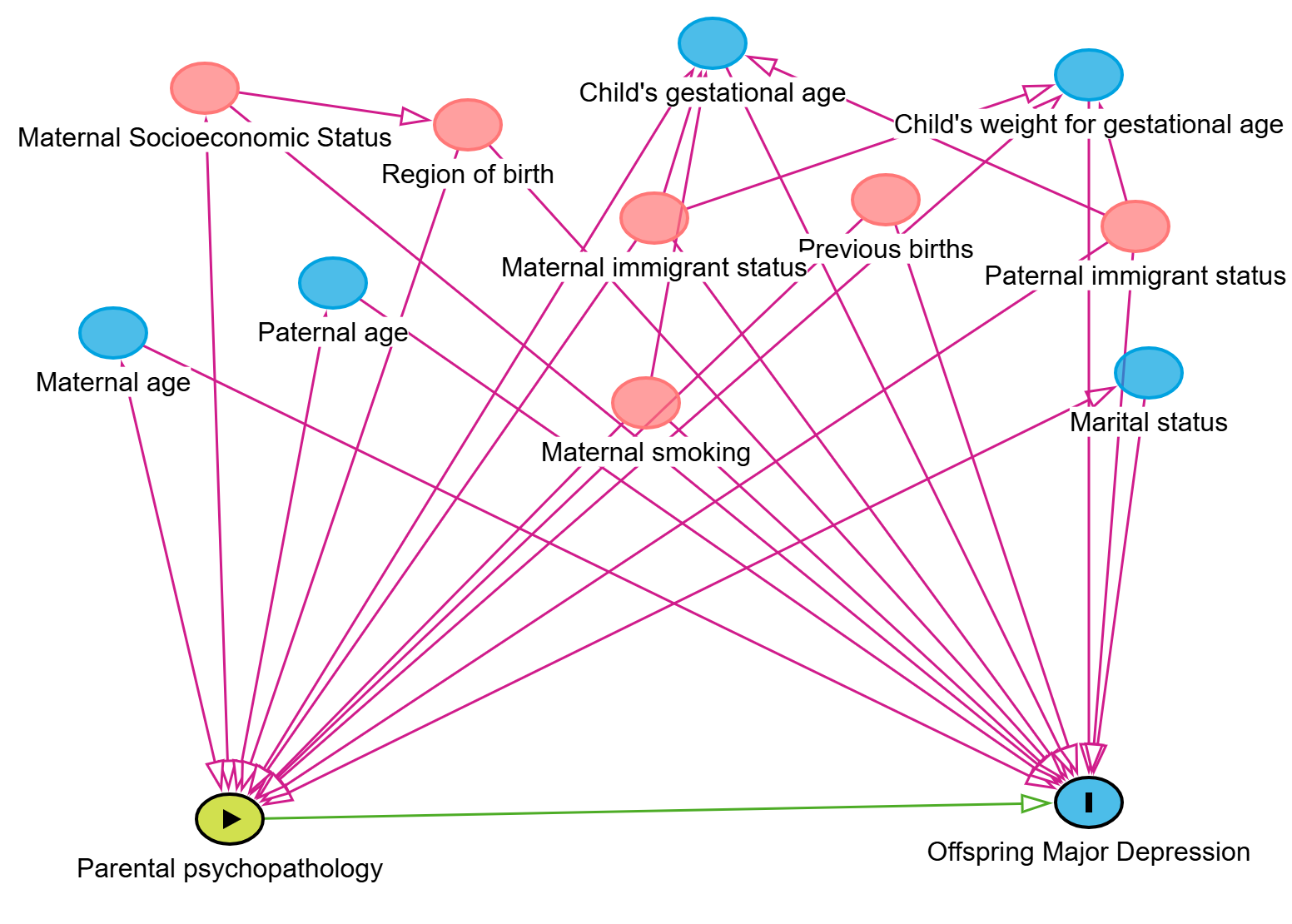


Figure S1. DAG diagram showing the role of covariates in the association between parental psychopathology and offspring major depression

Figure S2. Frequency distribution of a) maternal psychiatric diagnoses before and after child’s birth b) paternal psychiatric diagnoses before and after child’s birth. Zero (0) indicate child’s birth, negative values indicate before child’s birth and positive values indicate after child’s birth.


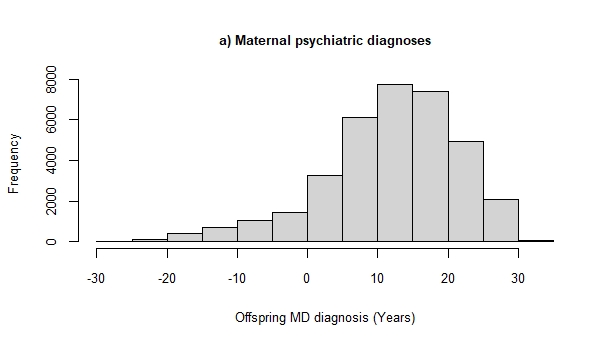

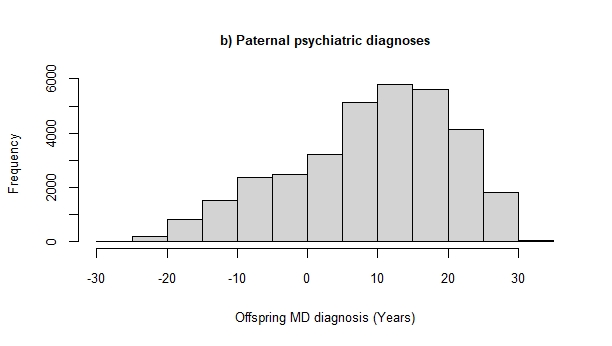

Supplement: Upadhyaya et al. supplementary material [file S0033291725000662sup001.docx]
